# Supplementary material for: On-demand male contraception via acute inhibition of soluble adenylyl cyclase
Source: Nat Commun. 2023 Feb 14;14:637. doi: 10.1038/s41467-023-36119-6 (PMC9929232; doi:10.1038/s41467-023-36119-6)
Supplement: Supplementary file 1 — Supplementary Information [file 41467_2023_36119_MOESM1_ESM.pdf]

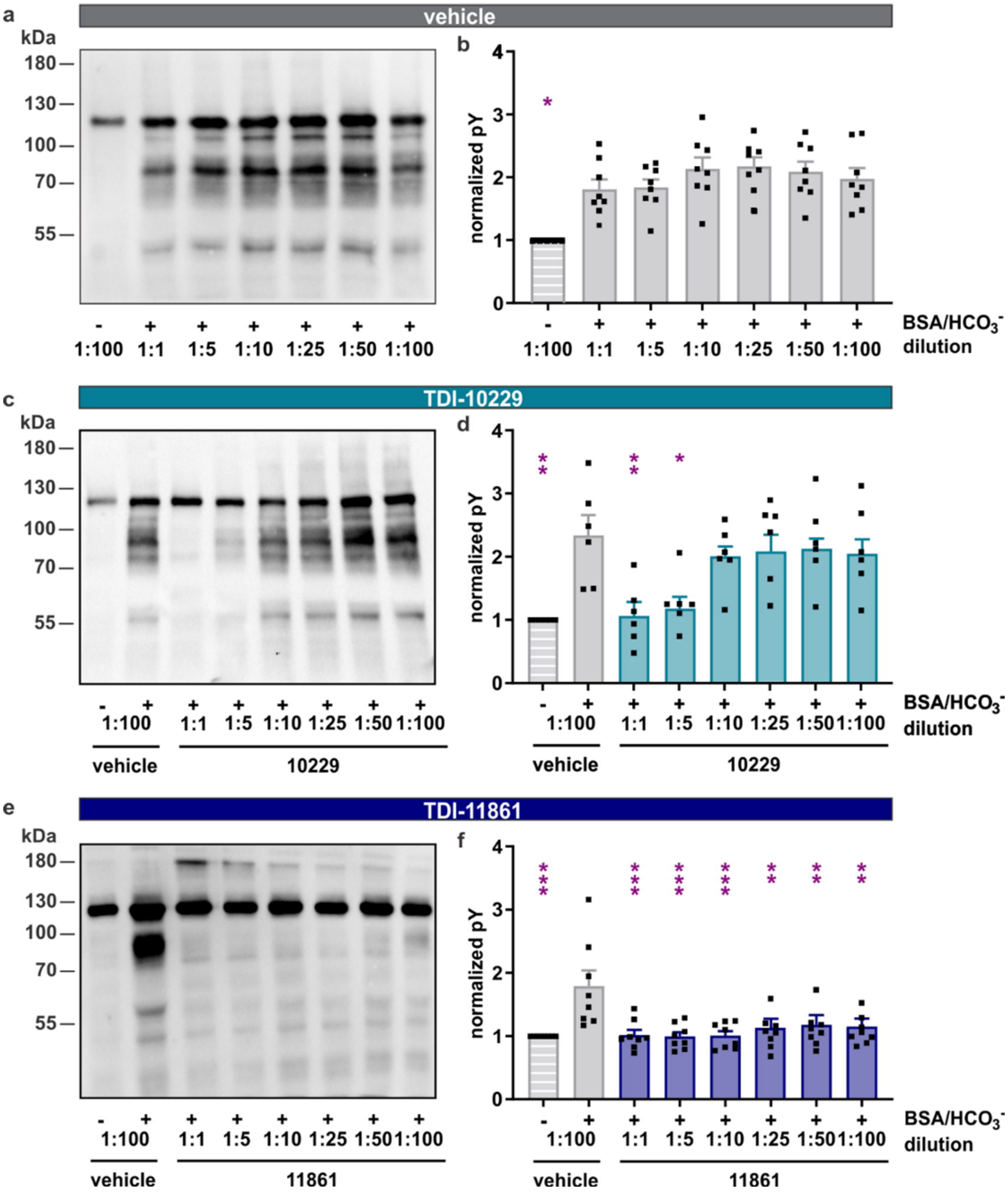

Supplementary Figure S1: Mouse sperm tyrosine phosphorylation is blocked after systemic exposure with sAC inhibitors

(a,c,e) Phosphorylation of tyrosine residues of epidydimal mouse sperm isolated one hour post injection (i.p.) with **(a)** vehicle, **(c)** 50 mg/kg TDI-10229 or **(e)** 50 mg/kg TDI-11861 after the indicated dilutions between 1:1 through 1:100 in inhibitor-free capacitating media. Shown are

representative Western Blots. **(b,d,f)** Quantitation of tyrosine residues of mouse sperm isolated from mice one hour post injection (i.p.) with **(b)** vehicle (grey), **(d)** 50 mg/kg TDI-10229 (teal) or **(f)** 50 mg/kg TDI-11861 (blue) after the indicated dilutions between 1:1 through 1:100 in inhibitor-free capacitating media. Tyrosine phosphorylation patterns were normalized to non-capacitated sperm (striped bars) from vehicle-injected controls; mean + SEM (vehicle n=8, TDI-10229 n=4, TDI-11861 n=8 with individual replicates indicates as symbols). Differences between conditions were analyzed using one-way ANOVA compared to the DMSO-treated capacitated control *P<0.05, **P< 0.01, ***P<0.001. Source data and uncut Western blot images are provided in the Source Data file, n=biological replicates.

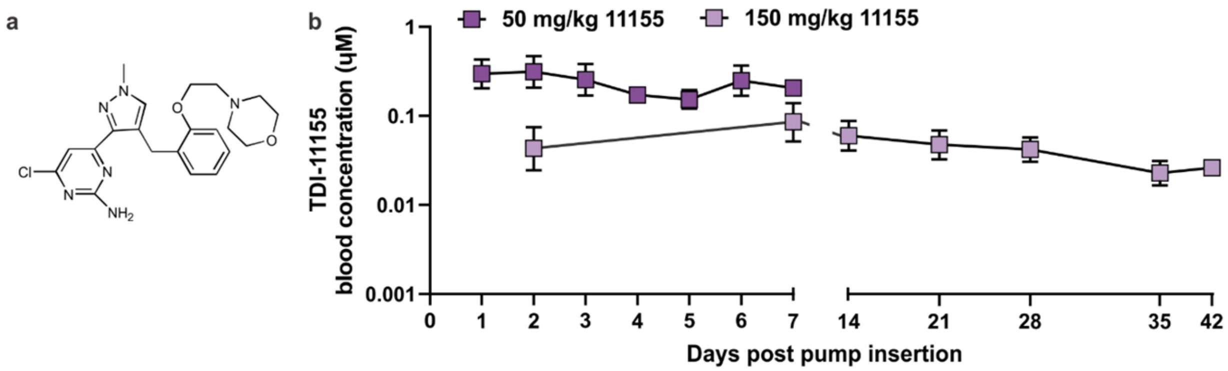

Supplementary Figure S2: Long-term systemic exposure of TDI-11155

(a) Molecular structure of TDI-11155. **(b)** TDI-11155 blood concentration quantified at the indicated time points post insertion of 7 day (Alzet pump model 2001) and 42 day (Alzet pump model 2006) osmotic minipumps filled with 50 mg/kg TDI-11155 (1 µl/hr release rate = 0.25 mg/kg/hr) and 150 mg/kg TDI-11155 (0.25 µl/hr release rate = 0.189 mg/kg/hr), respectively; mean ± SEM (n=3). Source data are provided in the Source Data file, n=biological replicates.

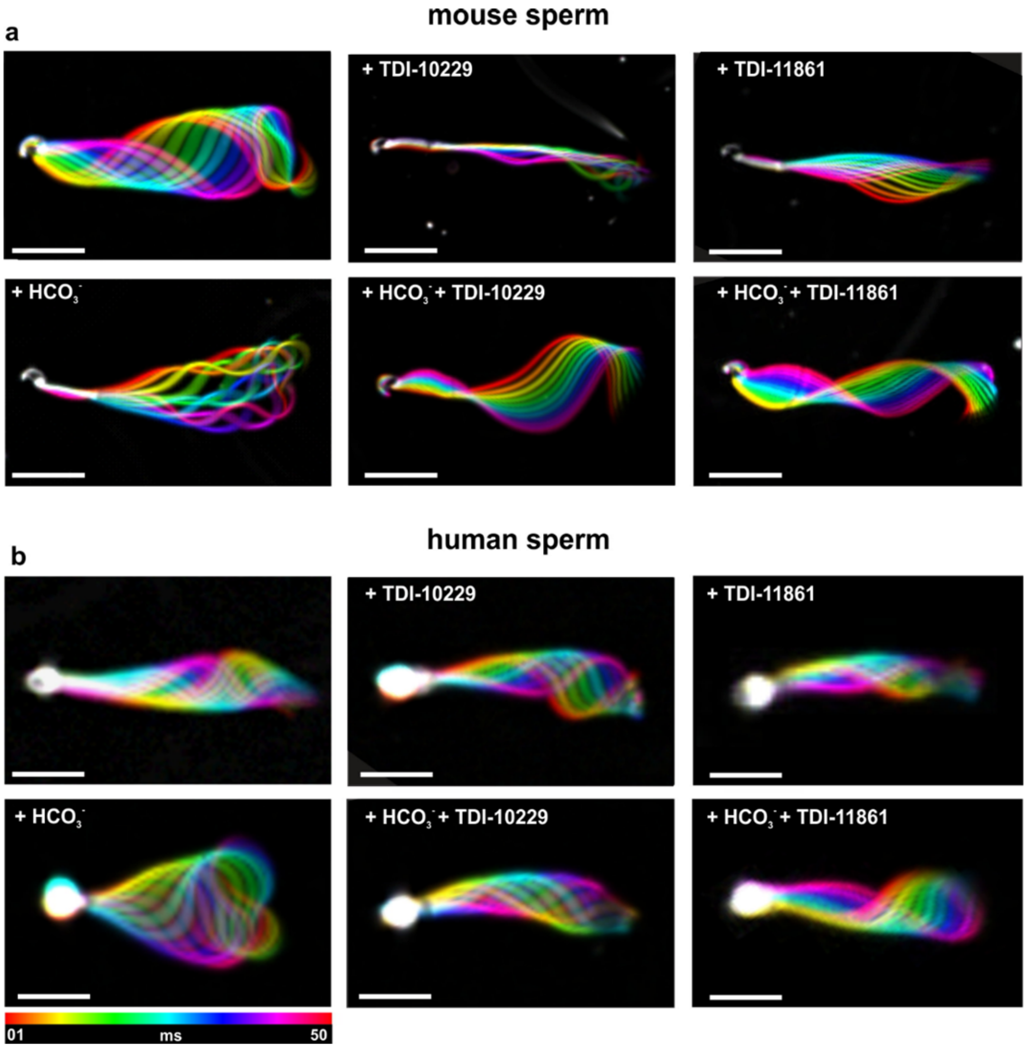

Supplementary Figure S3: Characterization of mouse and human sperm beating pattern after inhibition of sAC

(a,b) Flagellar waveform of **(a)** mouse and **(b)** human sperm in the absence or presence of 5 µM TDI-10229 or 100 nM TDI-11961 before and after stimulation with 25 mM $NaHCO_3$. Superimposed color-coded frames taken every 5 ms, illustrating one flagellar beat cycle; scale bar: 30 µm (mouse) or 15 µm (human). Shown are representative images of 3 independently replicated experiments (summarized in fig. 2 e,f).

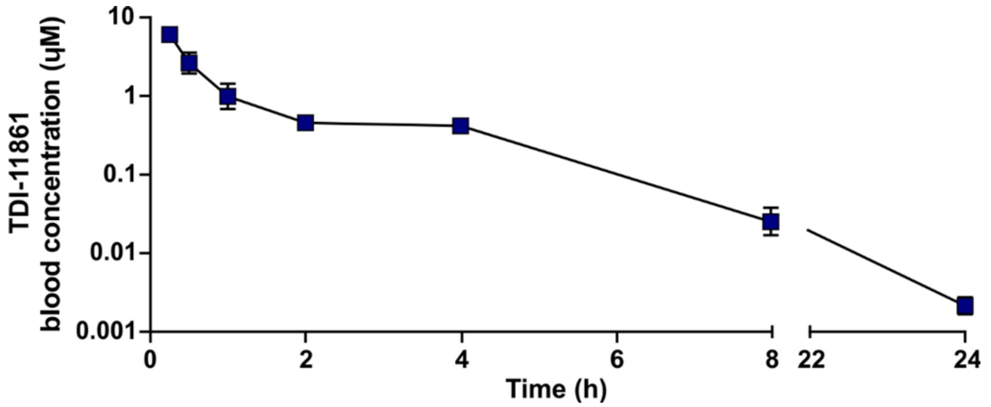

Supplementary Figure S4: Pharmacokinetic profile of orally delivered TDI-11861

TDI-11861 blood concentration quantified at the indicated time points post administration of 50 mg/kg TDI-11861 via oral gavage; mean ± SEM, n=3. Source data are provided in the Source Data file, n=biological replicates.

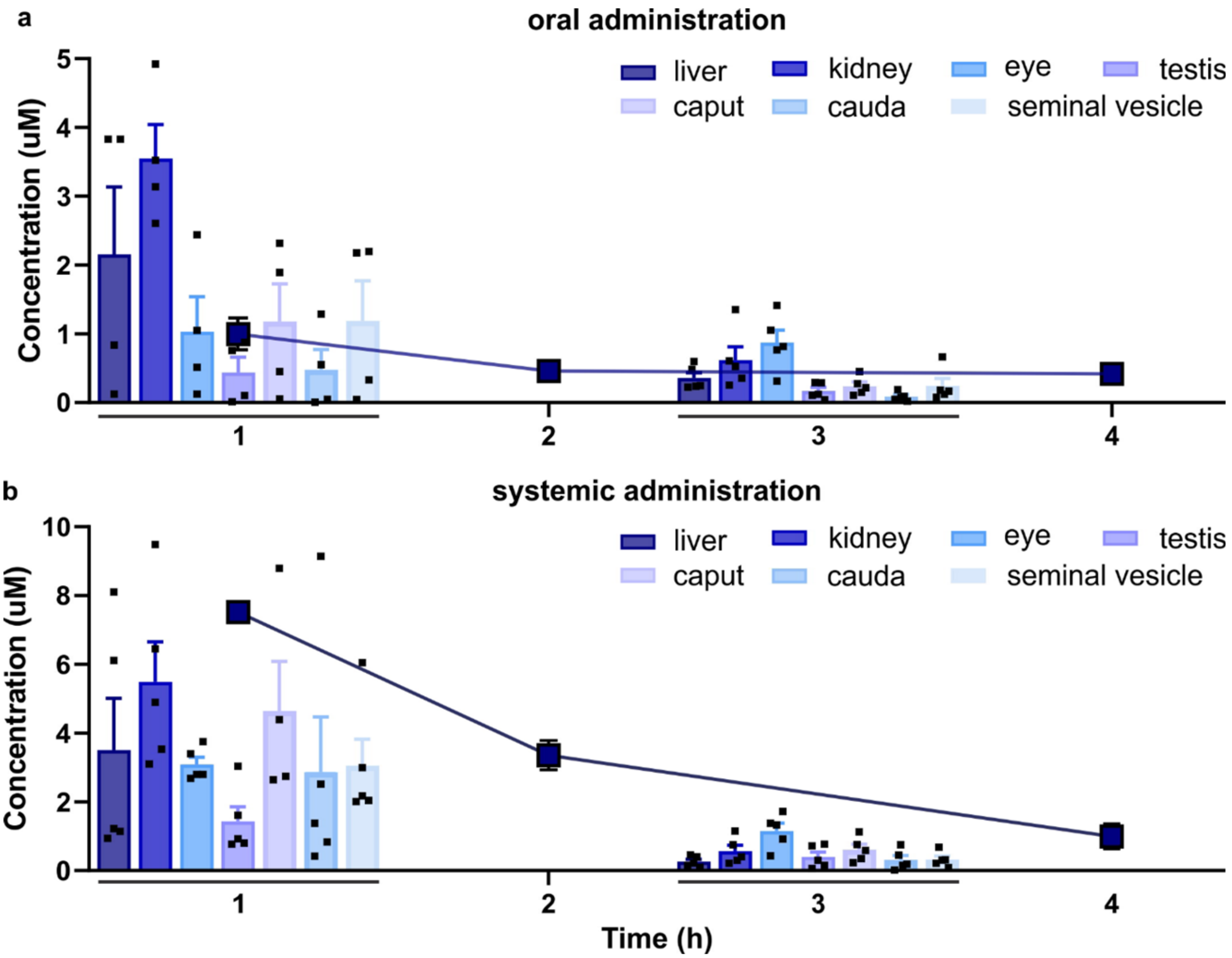

Supplementary Figure S5: TDI-11861 pharmacokinetics in reproductive and vital organs after oral and systemic delivery

(a,b) TDI-11861 concentration in liver (dark blue), kidney (navy blue), eye (sky blue), testis (violet), caput (periwinkle) and cauda (baby blue) epididymis, and seminal vesicle (light blue) one and three hours post (a) oral and (b) systemic administration of 50 mg/kg 11861 (bars) overlayed with respective TDI-11861 blood concentrations (squares), oral n=3, systemic tissues n=3; blood n=5 with individual replicates indicates as symbols. Source data are provided in the Source Data file, n=biological replicates.

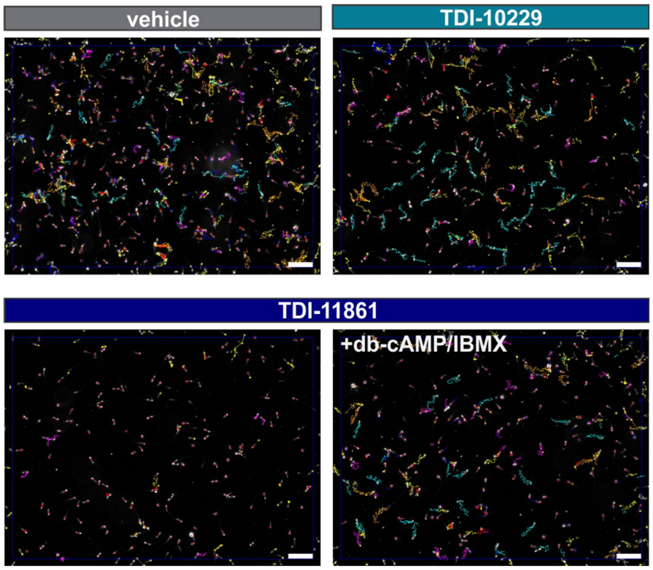

Supplementary Figure S6: Mouse sperm motility is blocked after oral administration of sAC inhibitors

Representative motility tracks of sperm isolated from male mice one h post oral administration with vehicle, 50 mg/kg TDI-10229 or 50 mg/kg TDI-11861 diluted 1:200 in inhibitor-free non-capacitating media. Motility of sperm isolated from TDI-11861-treated males was also assessed in the presence of 5 mM db-cAMP/500 µM IBMX. Shown are representative traces of at least 7 times independently replicated experiments (quantification is shown in fig. 3b), scale bar = 500 µM.

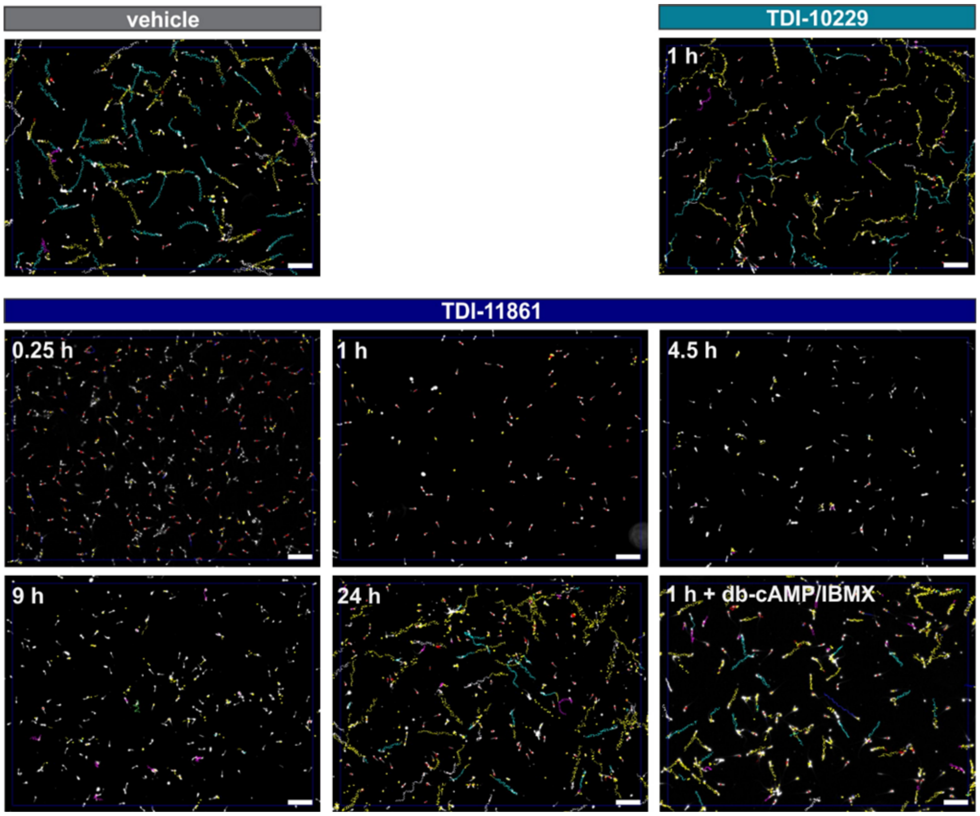

Supplementary Figure S7: Mouse sperm motility is blocked after systemic exposure with sAC inhibitors

Representative motility tracks of sperm isolated from male mice at the indicated time points post injection (i.p.) with vehicle, 50 mg/kg TDI-10229 or 50 mg/kg TDI-11861 diluted 1:200 in inhibitor-free non-capacitating media. Motility of sperm isolated after 1 h from TDI-11861-injected males was also assessed in the presence of 5 mM db-cAMP/500 µM IBMX. Shown are representative traces of at least 8 times independently replicated experiments (quantification is shown in fig. 3d), scale bar = 600 µM.

Supplementary Table S1: Histopathology report of mice chronically treated with TDI-11155

Light microscopy evaluation of H&E-stained sections from 6 animals (three treated with vehicle alone; three treated with TDI-11155) by a board-certified pathologist. There is no histomorphologic evidence of TDI-11155-related toxicity. There were no obvious differences or specific trends identified between vehicle and TDI-11155 treated animals, and most histologic findings were minimal to mild and also present in control animals.

Animal ID	vehicle_A	vehicle_B	vehicle_C	11155_A	11155_B	11155_C
Sex	Male	Male	Male	Male	Male	Male
Age	2 months	2 months	2 months	2 months	2 months	2 months
Species	Mouse	Mouse	Mouse	Mouse	Mouse	Mouse
Strain	C57BL/6J	C57BL/6J	C57BL/6J	C57BL/6J	C57BL/6J	C57BL/6J
Testis	Tubular degeneration/atrophy, 2, MF.	Tubular degeneration/atrophy, 2, MF. Tunica albuginea: lymphoplasmacytic and histiocytic infiltrates, 1, MF, perivascular.	Tubular degeneration/atrophy, 2-3, MF.	Tubular degeneration/atrophy, 2, MF.	Tubular degeneration/atrophy, 2, MF.	Tubular degeneration/atrophy, 2, MF.
Epididymis	Exfoliated germ cells, 1, MF. Epithelial karyomegaly, 2, MF.	Exfoliated germ cells, 1, MF. Epithelial karyomegaly, 2, MF.	Exfoliated germ cells, 1, MF. Epithelial karyomegaly, 2, MF. Lymphoplasmacytic and histiocytic infiltrates, 1, MF, interstitial.	Exfoliated germ cells, 1, MF. Epithelial karyomegaly, 2, MF.	Exfoliated germ cells, 1, MF. Epithelial karyomegaly, 2, MF.	Exfoliated germ cells, 1, MF. Epithelial karyomegaly, 2, MF.
Kidneys	Lymphoplasmacytic and histiocytic infiltrates, 1, MF, pelvic and perivascular.	Lymphoplasmacytic and histiocytic infiltrates, 1, MF, pelvic and perivascular. Tubular degeneration, cortical, 1, MF.	Lymphoplasmacytic and histiocytic infiltrates, 1, MF, pelvic and perivascular.	Lymphoplasmacytic and histiocytic infiltrates, 1, MF, pelvic.	Lymphoplasmacytic and histiocytic infiltrates, 1, MF, pelvic. Tubular degeneration, cortical, 1, MF. Tubular mineralization, 1, F, papillaRY.	Lymphoplasmacytic and histiocytic infiltrates, 1, MF, pelvic. Tubular basophilia, cortical, 1, F. Tubular mineralization, 1, F, papillaRY.
Liver	Extramedullary hematopoiesis, 1, MF, random.	Extramedullary hematopoiesis, 1, MF, random. Lymphoplasmacytic and histiocytic infiltrates, 2, MF, perivascular.	Extramedullary hematopoiesis, 1, MF, random.	Extramedullary hematopoiesis, 1, MF, random.	Extramedullary hematopoiesis, 1, MF, random. Lymphoplasmacytic, histiocytic, and neutrophilic hepatitis, 2, F, periportal.	Extramedullary hematopoiesis, 1, MF, random. Lymphoplasmacytic, histiocytic, and neutrophilic hepatitis, 1, F, midzonal.
Pancreas	N	Peri-pancreatic fat: lymphoplasmacytic and histiocytic infiltrates, 1, MF, perivascular.	N	N	N	N

Spleen	N	N	N	N	N	N
Eyes	N	N	N	N	N	N

N: normal, F: focal, MF: multifocal, 1: Minimal, 2: mild.

Testis:
Tubular degeneration/atrophy: This is a common incidental background finding in mice, and lesions are not restricted to a specific germ cell type or stage. The alteration generally presents as a small number of tubules with partial germ cell depletion and occasional degenerating germ cells with eosinophilic cytoplasm and nuclear condensation, multinucleated cells, cytoplasmic vacuolation, exfoliation, and a few atrophic tubular profiles lined only by Sertoli cells. Seminiferous tubule degeneration and/or atrophy can also be a manifestation of toxicologic injury to the testis, encompassing effects mediated through primary cytotoxicity causing Sertoli or germ cell injury, or through hormonal disruption, hypoxia, inflammation, or other effects. However, in this study, no obvious differences were identified between different groups.

Epididymis:
Exfoliated germ cells: This change is characterized by the presence of intact cells (often recognizable as round spermatids or spermatocytes) and/or cell debris intermixed with mature sperm within the ductular lumen. Normal young adult mice have a few sloughed cells in the epididymal lumen as a background finding. No associated degeneration or necrosis of the epididymal epithelium was identified in this study.
Epithelial karyomegaly: Atypical enlarged nuclei (karyomegaly) are most often seen in the epithelium of the ductus deferens, but can also be seen in other parts of the epididymis. Karyomegaly is characterized by epithelial cells that contain large, intensely basophilic staining nuclei that are often indented. It is generally seen as a background finding in mice.
Inflammatory cell infiltrates: These are common in C57BL/6 mice and are often of no pathologic significance. They are often composed of mononuclear cells with or without smaller numbers of neutrophils forming perivascular clusters or interspersed through the interstitium. The number of inflammatory cell foci increases with age and/or with the presence of infectious processes or disrupted immune system due to a variety of causes.

Kidneys:
Tubular degeneration: This is a nonspecific feature that can arise from a variety of etiologies involving hypoxia, disruption of ATP production, mitochondrial injury, free radical formation, peroxidation, or perturbed cell signaling, and it's often an early indicator of necrosis. In general, degeneration encompasses several morphologic changes and variable cell features in the renal epithelium associated with loss of viability, including tinctorial change, cell swelling with or without cytoplasmic vacuolation, blebbing, attenuated and/or fragmented cytoplasm and nucleus, cellular sloughing, and other alterations including repair.
Tubular basophilia: This represents tubular epithelial cells with basophilic cytoplasm, hypertrophy, and slightly increased nuclear:cytoplasmic ratio, but otherwise normal tubular profiles. This is the preferred term in the absence of any other evidence of degeneration or regeneration. Tubular basophilia may also be a sequel to degenerative conditions or represent excessive cellular turnover, but it also occurs as a normal feature in rodents in an increasing percentage with age.
Tubular mineralization: Mineralization of the medullary collecting ducts or papillae is a common spontaneous and minute background finding in rodents. In general, this most often represents intraluminal basophilic deposits of calcium that have no pathologic significance.
Inflammatory cell infiltrates: These are extremely common in C57BL/6 mice and are often of no pathologic significance. They are often composed of mononuclear cells with or without smaller numbers of neutrophils forming perivascular clusters or interspersed through the interstitium. The number of inflammatory cell foci increases with age and/or with the presence of chronic progressive nephropathy (CPN), infectious processes, or disrupted immune system due to a variety of causes.

Liver:
Extramedullary hematopoiesis: This is a common, nonspecific reactive/compensatory effect in response to increased hematopoietic demand secondary to a wide variety of systemic alterations, and it is also normally seen in the liver of young animals.
Hepatitis: This type of inflammatory changes in the liver are very commonly observed in experimentally naïve mice of commonly used strains. In most cases, the cause is not determined. Subclinical infection by murine norovirus and *Helicobacter* spp, have been reported to cause similar lesions. Most facilities at our institutions are not free of these infectious agents, but subclinical infection typically has no significant impact on research.
Inflammatory cell infiltrates: These are extremely common in C57BL/6 mice and are often of no pathologic significance. They are often composed of mononuclear cells with or without smaller numbers of neutrophils forming perivascular clusters or interspersed through the interstitium. The number of inflammatory cell foci increases with age and/or with the presence of infectious processes or disrupted immune system due to a variety of causes.

In summary, at the histologic level, upon light microscopy evaluation of the H&E-stained sections, there is no histomorphologic evidence of test article-related toxicity in the submitted tissues; no obvious differences or specific trends were identified between different groups; and most histologic findings were minimal to mild and also present in control animals.

Supplementary Table S2: Beat frequencies of mouse and human sperm in the presence and absence of sAC inhibitor

Beat frequencies were averaged over the distal 10 µm (for mouse sperm) or 5 µm (for human sperm) of the flagellum; mean ± SEM, n≥60 individual sperm from 3 different mice/donors.

	Basal (Hz)	+ HCO_3^- (Hz)
Mouse vehicle	26.3 ± 1.0	51.2 ± 2.0
Mouse + TDI-10229	10.4 ± 0.8	11.7 ± 1.8
Mouse + TDI-11861	14.8 ± 1.0	22.7 ± 1.6
Human vehicle	17.8 ± 0.6	26.4 ± 0.6
Human + TDI-10229	12.2 ± 0.8	14.5 ± 0.6
Human + TDI-11861	11.4 ± 1.3	11.0 ± 0.8
