# Supplementary material for: On-demand male contraception via acute inhibition of soluble adenylyl cyclase
Source: Nat Commun. 2023 Feb 14;14:637. doi: 10.1038/s41467-023-36119-6 (PMC9929232; doi:10.1038/s41467-023-36119-6)
Supplement: Supplementary file 3 — Description of Additional Supplementary Files [file 41467_2023_36119_MOESM3_ESM.pdf]

Description of Additional Supplementary Files

File Name: Supplementary Movie 1
Description: Flagellar motility of mouse sperm in the presence of 5 µM TDI-10229 or 100 nM TDI-11861 before and after stimulation with 25 mM $NaHCO_3$.

File Name: Supplementary Movie 2
Description: Flagellar motility of human sperm in the presence of 5 µM TDI-10229 or 100 nM TDI-11861 before and after stimulation with 25 mM $NaHCO_3$.

File Name: Supplementary Movie 3
Description: Motility of mouse sperm isolated from male mice one hour post oral administration of vehicle, 50 mg/kg TDI-10229, or 50 mg/kg TDI-11861. Motility of sperm isolated from epididymis of male mice was also assessed in the presence of db-cAMP/IBMX.

File Name: Supplementary Movie 4
Description: Motility of sperm isolated from epididymis of male mice injected (i.p.) with vehicle control.

File Name: Supplementary Movie 5
Description: Motility of sperm isolated from epididymis of male mice one hour post injection (i.p.) with 50 mg/kg TDI-10229.

File Name: Supplementary Movie 6
Description: Motility of sperm isolated from epididymis of male mice one hour post injection (i.p.) with 50 mg/kg TDI-11816. Motility of sperm isolated after 1 h was also assessed in the presence of db-cAMP/IBMX.

File Name: Supplementary Movie 7
Description: Motility of ejaculated mouse sperm isolated from the uterus of receptive females one hour post copulation from male mice injected (i.p) with vehicle or 50 mg/kg TDI-11816.
