# Supplementary material for: On-demand male contraception via acute inhibition of soluble adenylyl cyclase
Source: Nat Commun. 2023 Feb 14;14:637. doi: 10.1038/s41467-023-36119-6 (PMC9929232; doi:10.1038/s41467-023-36119-6)
Supplement: Supplementary file 11 — Reporting Summary [file 41467_2023_36119_MOESM11_ESM.pdf]

nature portfolio

Corresponding author(s): Jochen Buck & Lonny R. Levin

Last updated by author(s): Dec 14, 2022

Reporting Summary

Nature Portfolio wishes to improve the reproducibility of the work that we publish. This form provides structure for consistency and transparency in reporting. For further information on Nature Portfolio policies, see our Editorial Policies and the Editorial Policy Checklist.

Please do not complete any field with "not applicable" or n/a. Refer to the help text for what text to use if an item is not relevant to your study.
For final submission: please carefully check your responses for accuracy; you will not be able to make changes later.

Statistics

For all statistical analyses, confirm that the following items are present in the figure legend, table legend, main text, or Methods section.

n/a | Confirmed

The exact sample size (n) for each experimental group/condition, given as a discrete number and unit of measurement

A statement on whether measurements were taken from distinct samples or whether the same sample was measured

repeatedly The statistical test(s) used AND whether they are one- or two-sided

Only common tests should be described solely by name; describe more complex techniques in the Methods section.

A description of all covariates tested

A description of any assumptions or corrections, such as tests of normality and adjustment for multiple comparisons

A full description of the statistical parameters including central tendency (e.g. means) or other basic estimates (e.g. regression coefficient) AND variation (e.g. standard deviation) or associated estimates of uncertainty (e.g. confidence intervals)

For null hypothesis testing, the test statistic (e.g. F, t, r) with confidence intervals, effect sizes, degrees of freedom and P value noted

Give P values as exact values whenever suitable.

For Bayesian analysis, information on the choice of priors and Markov chain Monte Carlo settings

For hierarchical and complex designs, identification of the appropriate level for tests and full reporting of outcomes

Estimates of effect sizes (e.g. Cohen's d, Pearson's r), indicating how they were calculated

Our web collection on statistics for biologists contains articles on many of the points above.

Software and code

Policy information about availability of computer code

Data collection	Sperm population motility was assessed via Computer Assisted Sperm Analysis (CASA) Hamilton–Thorne digital image analyzer (IVOS II, Hamilton Thorne Research, Beverly, MA) with the following parameters: 30 frames, frame rate: 60 Hz, cell size: 30–170 µm2. Confocal images were collected using ZEN Black version 14.0.27.201.
Data analysis	Statistical relevance was calculated in PRISM (Version 9). Sperm motility for individual attached sperm was quantitated using SpermQ software version 1.0.8 (Hansen et al., Cells 2018) and for populations using CASA.

For manuscripts utilizing custom algorithms or software that are central to the research but not yet described in published literature, software must be made available to editors and reviewers. We strongly encourage code deposition in a community repository (e.g. GitHub). See the Nature Portfolio guidelines for submitting code & software for further information.

Data

Policy information about availability of data

All manuscripts must include a data availability statement. This statement should provide the following information, where applicable:

- Accession codes, unique identifiers, or web links for publicly available datasets
- A description of any restrictions on data availability
- For clinical datasets or third party data, please ensure that the statement adheres to our policy

Primary data are included in source data file. Requests for materials should be addressed to Drs. Jochen Buck (email: jobuck@med.cornell.edu) and Lonny R. Levin (email: llevin@med.cornell.edu).

Human research participants

Policy information about studies involving human research participants and Sex and Gender in Research.

Reporting on sex and gender	Only men can be sperm donors
Population characteristics	Donors reflect the ethnic makeup of the Weill Cornell Medicine community and included healthy males ages 25 – 35. Only samples that met the WHO 2010 criteria for normal semen parameters (ejaculated volume ≥ 1.5 mL, sperm concentration ≥ 15 million/mL, motility ≥ 40%, progressive motility ≥32%, normal morphology ≥ 4%) were included in this study.
Recruitment	Volunteers responded to flyers (approved by WCM IRB) placed at random around the Weill Cornell Medical College and Graduate School of Medical Sciences. Since volunteers were unpaid, the only bias present in the volunteer population would be those interested in aiding in developing a male contraceptive. This bias should have no impact on the study.
Ethics oversight	Samples of human semen were obtained from healthy volunteers with their prior written consent following a protocol approved by Weill Cornell Medicine's Institutional Review Board (IRB 21-03023495).

Note that full information on the approval of the study protocol must also be provided in the manuscript.

Field-specific reporting

Please select the one below that is the best fit for your research. If you are not sure, read the appropriate sections before making your selection.

☒ Life sciences ☐ Behavioural & social sciences ☐ Ecological, evolutionary & environmental sciences

For a reference copy of the document with all sections, see nature.com/documents/nr-reporting-summary-flat.pdf

Life sciences study design

All studies must disclose on these points even when the disclosure is negative.

Sample size	Number of repeats for In vitro and ex vivo experiments were based upon previous published studies and were consistent with published reports, see for example Balbach et al MHR 2021. For In vivo mating, the number of pairings was approximately 50 to ensure 'double digit' pregnancies in our vehicle control.
Data exclusions	No mouse data were excluded; For human sperm, only samples that met the WHO 2010 criteria for normal semen parameters (ejaculated volume ≥ 1.5 mL, sperm concentration ≥ 15 million/mL, motility ≥ 40%, progressive motility ≥32%, normal morphology ≥ 4%) were included in this study.
Replication	All data represent averages or means of experiments independently replicated at least 5 times (i.e., on different days using samples from distinct individual mice or men)..
Randomization	Male mice for sperm collection were selected at random and samples from each individual were separated randomly for use in treated versus control experiments. Female mice were chosen based upon when they entered estrus. For human sperm, samples were collected and used at random from our current pool of seven male donors. For ex vivo motility and mating experiments, males (and females in estrus) were assigned into vehicle or compound treated groups at random.
Blinding	Blinding for mating studies was not relevant, as there is no possibility of experimenter bias in identifying pregnancy. For histology, the pathologist was blinded prior to analysis.

Reporting for specific materials, systems and methods

We require information from authors about some types of materials, experimental systems and methods used in many studies. Here, indicate whether each material, system or method listed is relevant to your study. If you are not sure if a list item applies to your research, read the appropriate section before selecting a response.

Materials & experimental systems

n/a	Involved in the study	
☐	☒	Antibodies
☐	☒	Eukaryotic cell lines
☒	☐	Palaeontology and archaeology
☐	☒	Animals and other organisms
☒	☐	Clinical data
☒	☐	Dual use research of concern
☒	☐	

Methods

n/a	Involved in the study	
☒	☐	ChIP-seq
☒	☐	Flow cytometry
☒	☐	MRI-based neuroimaging

Antibodies

Antibodies used	For functional sperm assays, anti-phosphotyrosine (anti-PY) monoclonal antibody (clone 4G10) was obtained from Millipore (05-321).
Validation	Each batch purchased was validated using previously positive (i.e., capacitated sperm from WT mice) and negative (i.e., non-capacitated sperm from WT mice) samples.

Eukaryotic cell lines

Policy information about cell lines and Sex and Gender in Research

Cell line source(s)	4-4 cells was generated in our laboratory. It is a stable cell line which overexpresses rat sAC in HEK293 cells.
Authentication	We routinely confirm its identity using a combination of Western Blotting, diagnostic PCR, and biochemical cyclase assay.
Mycoplasma contamination	We regularly test to ensure all cell culture in our laboratory remain mycoplasma free.
Commonly misidentified lines (See ICLAC register)	No commonly misidentified lines were used in this study.

Animals and other research organisms

Policy information about studies involving animals; ARRIVE guidelines recommended for reporting animal research, and Sex and Gender in Research

Laboratory animals	Adult C57BL/6J male and female mice (8-10 weeks old) were purchased from Jackson Laboratories and allowed to acclimatize before use.
Wild animals	No Wild animals were used in these studies.
Reporting on sex	Sperm were collected from males; matings involved both males and females.
Field-collected samples	No field-collected samples were used in these studies.
Ethics oversight	Animal experiments were approved by Weill Cornell Medicine's Institutional Animal Care and Use Committee (IACUC).

Note that full information on the approval of the study protocol must also be provided in the manuscript.

This checklist template is licensed under a Creative Commons Attribution 4.0 International License, which permits use, sharing, adaptation, distribution and reproduction in any medium or format, as long as you give appropriate credit to the original author(s) and the source, provide a link to the Creative Commons license, and indicate if changes were made. The images or other third party material in this article are included in the article's Creative Commons license, unless indicated otherwise in a credit line to the material. If material is not included in the article's Creative Commons license and your intended use is not permitted by statutory regulation or exceeds the permitted use, you will need to obtain permission directly from the copyright holder. To view a copy of this license, visit http://creativecommons.org/licenses/by/4.0/
